# Anti-correlated cortical networks arise from spontaneous neuronal dynamics at slow timescales

**DOI:** 10.1038/s41598-017-18097-0

**Published:** 2018-01-12

**Authors:** Nathan X. Kodama, Tianyi Feng, James J. Ullett, Hillel J. Chiel, Siddharth S. Sivakumar, Roberto F. Galán

**Affiliations:** 1 Department of Electrical Engineering and Computer Science, Case Western Reserve University, Cleveland, Ohio 44106 USA; 2 Department of Biology, Case Western Reserve University, Cleveland, Ohio 44106 USA

## Abstract

In the highly interconnected architectures of the cerebral cortex, recurrent intracortical loops disproportionately outnumber thalamo-cortical inputs. These networks are also capable of generating neuronal activity without feedforward sensory drive. It is unknown, however, what spatiotemporal patterns may be solely attributed to intrinsic connections of the local cortical network. Using high-density microelectrode arrays, here we show that in the isolated, primary somatosensory cortex of mice, neuronal firing fluctuates on timescales from milliseconds to tens of seconds. Slower firing fluctuations reveal two spatially distinct neuronal ensembles, which correspond to superficial and deeper layers. These ensembles are anti-correlated: when one fires more, the other fires less and vice versa. This interplay is clearest at timescales of several seconds and is therefore consistent with shifts between active sensing and anticipatory behavioral states in mice.

## Introduction

Spontaneous activity in brain circuits provides a means to investigate functional connections between neurons or brain areas, which is crucial to understand how the brain processes information^1^. Functional connections indeed constrain the repertoire of activity patterns that neural networks can display^2^. Isolated brain circuits *in vitro* display spontaneous activity in the form of heterogeneous, spatiotemporal patterns^3^. These patterns often obey power laws^4,5^, a signature of complex systems at criticality, that is, on the verge of becoming unstable with exponentially growing fluctuations. However, it remains unclear how local brain circuits organize into operational modes and whether the functional connections between neurons are different across timescales.

We set out to investigate these questions in local neocortical circuits, using planar microelectrode arrays (MEAs) on acute brain slices. This technology has been previously applied to organotypic cultures^4,5^ and to record local field potentials from acute brain slices^6–8^. Spontaneous spiking activity *in vitro* may be achieved through a series of recently established brain slicing, slice incubation, and well-oxygenated perfusion solutions^9,10^. The motivation for investigating spontaneous activity in neocortical circuits is twofold. First, spontaneous activity within the neocortex dominates over sensory-evoked activity^11^ and reflects the disproportionately high anatomical connections between neocortical neurons versus feedforward sensory inputs^12,13^. Second, spontaneous circuit events observed with calcium imaging or single cell approaches have been shown to display a multiplicity of population sequences and neuronal firing characteristics, suggesting that the operational organization of neocortical ensembles is dynamic and diversely constituted^3,14^. In contrast, stimulus- or drug-evoked responses may not reveal as much of this diversity in operational organization.

Here, we focus on the isolated primary somatosensory cortex (S1), whose dynamics have been extensively characterized in the exploratory modes of behaving animals. At a brain systems level, exploratory modes are intimately linked to intracortical loops as well as subcortical relay structures, such as the thalamic nuclei^15,16^. Early activation of deep layers correlates with anticipatory behavior, whereas early activation of superficial layers correlates with active sensing^17^. However, the neocortical circuit’s capacity for diverse and organized spontaneous dynamics—in the absence of subcortical connections and arising solely from its intrinsic interconnections—has not been explored.

Several specific discoveries were made in the course of this investigation. (1) Although some individual neurons fired only transiently or turned on and off, the ensemble activity was, on the whole, sustained across the neuronal network. (2) Diversity in firing was vast, both across neurons and in the set of firing patterns from a single neuron. (3) Consistent with this diversity, a fluctuation scaling law and a set of frequency bands emerged, pointing to the existence of multiple timescales. (4) Functional connectivity, estimated as cross-correlations in neuronal firing, depended critically on the timescale of interest: while weak, sparse, and positive correlations existed at fast timescales, correlations characteristic of two competing networks progressively emerged at slow timescales. (5) These anti-correlated networks were heterogeneously constituted and localized to superficial and deeper layers of the cortical anatomy.

## Results

**Figure 1:**
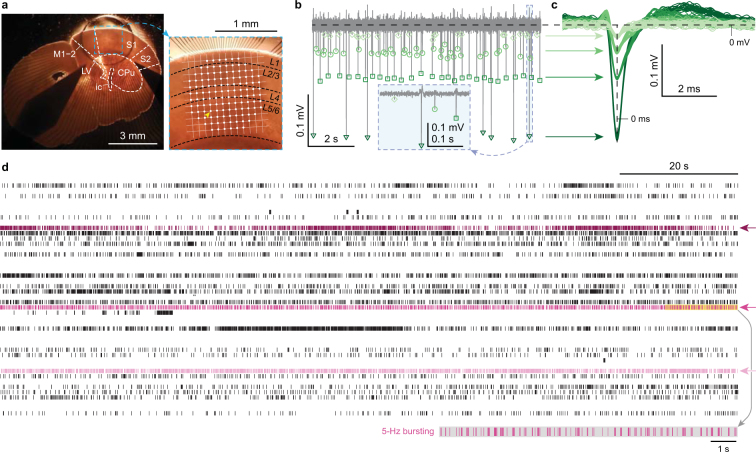
From multichannel electrophysiology to high-dimensional neuronal activity. (**a**) Coronal slice of the murine somatosensory cortex; a high density microelectrode array (white dots) was tangentially aligned with the pia. S1, primary somatosensory cortex; S2, secondary somatosensory cortex; M1–2, primary and secondary motor cortices; LV, lateral ventricle; ic, internal capsule; CPu, caudoputamen; L1–6, cortical layers. (**b**) Extracellular action potentials recorded from the L5/6 electrode marked by the yellow arrowhead in **a**. Inset, high temporal resolution of recording enables precise spike detection. (**c**) Action potential waveforms from a single electrode cluster into different groups, corresponding to different neurons. (**d**) Raster plot of spiking patterns from multiple (*n* = 46) neurons recorded simultaneously reveals diversity both in time and across neurons. Magenta arrows highlight a neuron with slowly modulated firing (dark magenta), a neuron bursting at 5 Hz (medium magenta), and a fast tonically firing neuron (light magenta). Inset, neuron bursting at 5 Hz.

Using high-density MEAs, we have recorded spontaneous network activity in murine S1 for 10–30 minutes *in vitro* (Fig. 1a). Stable slice attachment resulted in reliable recording (up to 10 dB signal-to-noise ratio, Fig. 1b) of extracellular spikes, and dense spatiotemporal sampling allowed for the resolution of roughly two hundred neurons (190 ± 9 neurons, mean ± s.e.m. across seven mice) simultaneously from all cortical layers (Fig. 1a). Neurons were semi-automatically identified based on the consistency of their waveforms with the aid of a spike-sorting algorithm^18^, as described in Methods. Figure 1c illustrates this procedure for multiple action potentials recorded from a single electrode in layer 5/6. Their waveforms cluster into four different groups with varying widths and amplitudes, where each group represents a different neuron. The timing of the spikes can then be resolved for all neurons across all electrodes (Fig. 1d).

### Spectra of spontaneous firing reveal multiple timescales

**Figure 2:**
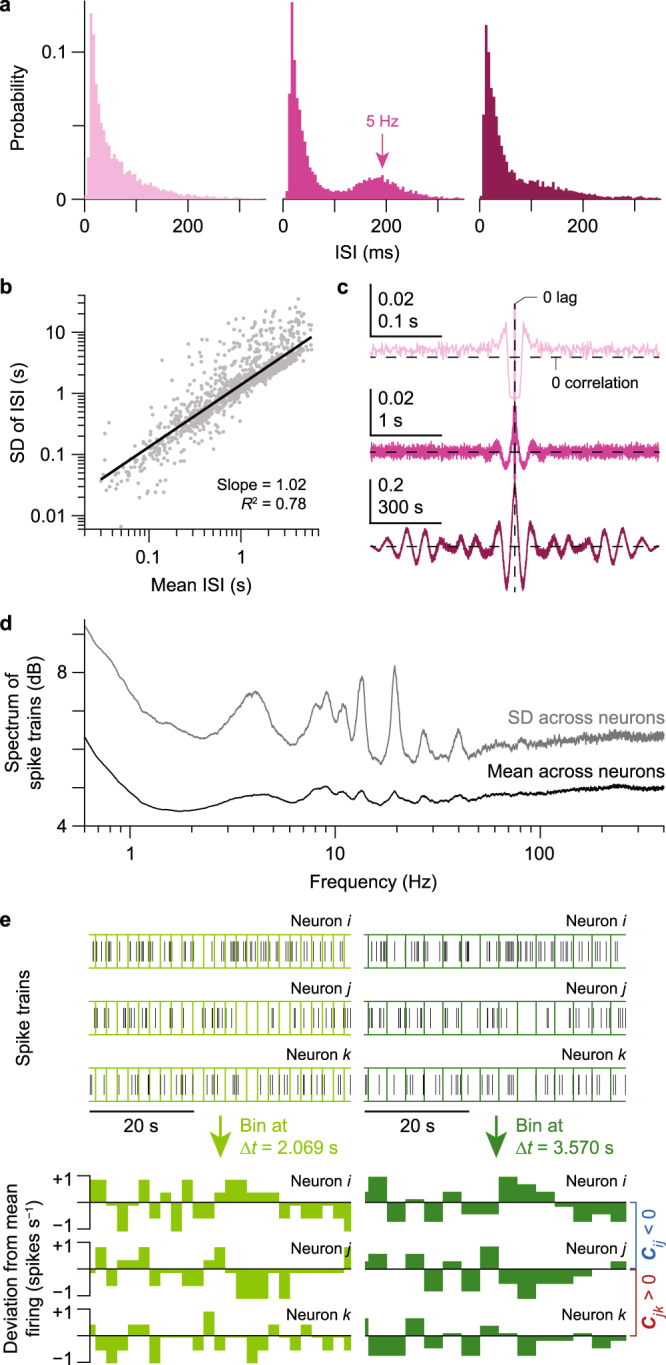
Diversity in spontaneous neural activity. (**a**) Interspike interval (ISI) histograms of three neurons highlighted in Fig. 1d from a single mouse. Multimodality in the distributions, such as the 5-Hz peak indicated in the second histogram, points to bursting patterns. (**b**) Power law between mean ISI and standard deviation (standard deviation) of ISI (*n* = 1,196 neurons from seven mice) with an exponent of ~1 demonstrates that spontaneous firing is super-Poissonian. Solid black line shows linear fit between log_10_ of mean ISI and log_10_ of standard deviation of ISI; *R*^2^ value of this fit is reported (*P* < 2 × 10^−308^, linear regression), and slope of this line corresponds to the exponent of the power law. (**c**) Auto-correlograms of spike trains from three selected neurons display oscillations over time, indicating firing rate modulation at multiple timescales (note the different time axes). (**d**) Mean spectrum of spike trains (black trace) and its standard deviation (gray trace) across experiments (*n* = 1,196 neurons from seven mice) reveal multiple frequency bands of spontaneous activity. (**e**) Spike train binning for three representative neurons from the same experiment, for two different bin widths (∆*t*). Green traces, time series of mean-subtracted binned spike counts normalized by ∆*t*. Series from neurons *i* and *j* are negatively correlated, and those from neurons *j* and *k* are positively correlated.

Despite considerable variations in firing pattern from neuron to neuron (Fig. 1d), statistical regularities emerged over multiple timescales. For instance, fast rhythmic bursting, as well as high frequency tonic firing, were commonly observed (Fig. 2a). Interestingly, these classes of neurons also expressed slower fluctuations in their firing rates (Fig. 2b). A single neuron’s spontaneous spiking in the network could be progressively evaluated across five orders of magnitude via its auto-correlogram (Fig. 2c and Movie S1). In spite of this diversity, both across timescales and across neurons, all neurons (*n* = 1,196 neurons from seven mice) followed a power law relationship between the mean and standard deviation of their interspike intervals (ISI; Fig. 2b), a phenomenon characteristic of complex systems known as *fluctuation scaling* in neuronal firing^19^ or Taylor’s Law in ecology. If spontaneous firing were completely random with a Poisson distribution, the standard deviation would be on average proportional to the square root of the mean. In contrast, spontaneous firing was observed to have a *super-Poissonian* distribution, with the standard deviation being proportional to the mean itself. Moreover, the spectrum of all neuronal spiking (*n* = 1,196 neurons from seven mice) further pointed to the coexistence of activity at multiple timescales and defined frequency bands (Fig. 2d) commonly found in the cerebral cortex^20^.

### Functional connectivity reveals the progressive emergence of anti-correlated networks from fast to slow timescales

We then investigated how neurons interact with each other across timescales. Typically, continuous signals may be filtered in certain frequency bands of interest. Because of the binary and discrete nature of the spike trains, standard digital filtering was not a suitable option. Instead, we treated the spike trains as count processes^21,22^, by dividing the time axis into consecutive bins of width ∆*t*, and counting the spikes in each bin (Fig. 2e, top). Thus, for each neuron at a given ∆*t*, we obtained a time series of the spike count, which allowed us to investigate firing fluctuations and their correlations across neurons (Fig. 2e, bottom). A multiscale correlation of progressively binned signals revealed functionally connected populations at logarithmically-spaced time scales, each corresponding to a well-defined bin width ∆*t*.

**Figure 3:**
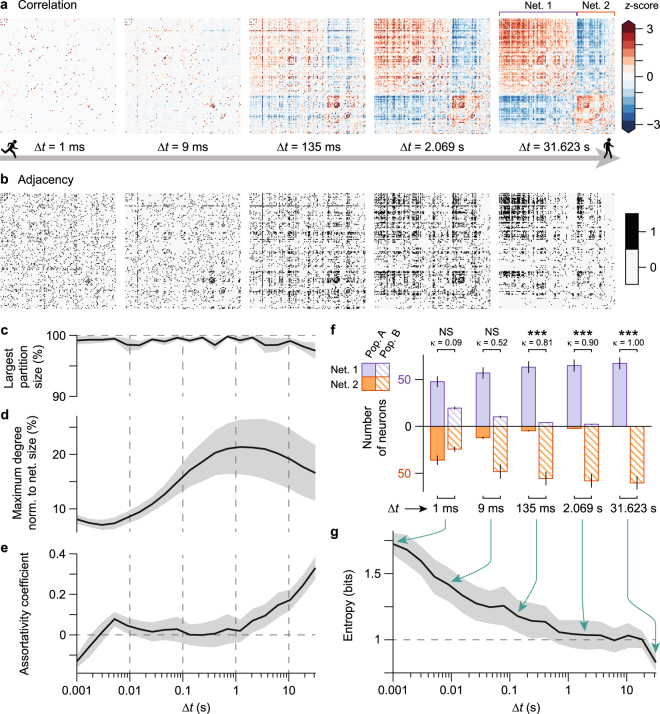
Network connectivity and graph analyses reveal anti-correlated networks at slow timescales. (**a**) Correlation matrices of binned spike counts of *n* = 149 neurons from a single mouse, at five selected timescales (∆*t*). Correlation values were normalized by their *z*-score at each timescale. Towards slower timescales, neuronal connections are stronger and denser, and shape up into two mutually exclusive, anti-correlated networks (Net. 1, Net. 2). (**b**) Binary adjacency matrices computed as either highly significant (1) or non-significant (0) pairwise correlations from correlation matrices in a at each timescale. Adjacency matrix at each timescale thus defines an undirected graph of neuronal connectivity (see *Connectivity and graph analysis* in Methods). (**c**–**e**) Graph theoretic measures computed from adjacency matrices in **b**. Norm., normalized. (**f**) At each timescale, the anti-correlated component analysis (ACA, see Methods) identifies two neuronal populations (Pop. A, Pop. B) that overlap with the two anti-correlated networks (Net. 1, Net. 2) respectively. The overlap decreases at faster timescales. NS, not significant; ***P < 0.001 (Cohen’s kappa test for inter-rater agreement). (**g**) Entropy of the contingency table in **f** demonstrates increasing order at progressively slower timescales. Largest entropy is attained at the fastest timescale of ∆*t* = 1 ms. Data are shown as mean ± s.e.m. across six mice in **c**–**g**.

On slow timescales (∆*t* = 100 ms and longer), anti-correlated networks emerged in spite of sparse and weakly positive correlations at faster timescales (down to ∆*t* = 1 ms). Fluctuations in firing rate and their correlations (Fig. 3a) became stronger, denser, and increasingly ordered at slow timescales, clearly revealing two anti-correlated networks (Movie S2): neurons within one network were positively correlated with other neurons in the same network, but were negatively correlated with neurons in the other network. This correlation structure was highly consistent across experiments (Fig. S1) and disappeared when the binned spike counts were randomly shuffled (Fig. S1, bottom row).

Highly significant correlations between neuronal pairs were used to construct a binary adjacency matrix, thus defining a graph of neuronal connectivity. The adjacency matrices across timescales (Fig. 3b) reinforce the notion that network connectivity became denser at slow timescales. The increased network connectivity at slow timescales was also highly consistent across experiments (Fig. S2).

Several graph theoretic measures, quantified from the adjacency matrices, revealed how the networks changed across timescales. First, the fraction of connected neurons relative to the number of identified neurons—the largest graph partition—was 95% or greater across all time scales (Fig. 3c). The largest degree—the number of connected neurons to a given neuron—changed non-monotonically across timescales and attained a maximum at an intermediate timescale of ∆*t* = 2 s (Fig. 3d). In contrast, the assortativity of the network—the preference of neurons to connect to neurons of the same degree—was non-negligible only on slow timescales (Fig. 3e), which is consistent with the emergence of highly structured networks.

Although the anti-correlated networks were clearest at slow timescales, their emergence was readily apparent on the order of ∆*t* ∼100 ms (Fig. 3a). This led us to investigate how the neurons in the anti-correlated networks progressively blended with each other from slow to fast timescales (right to left in Fig. 3a). The propensity of both networks to blend with one another at fast time scales was investigated to answer whether or not these networks were unique to slow timescales. To this end, we developed an algorithm to identify the two most anti-correlated, mutually exclusive networks given an arbitrary correlation matrix ***C***^23^. We refer to this algorithm as anti-correlated component analysis (ACA; see Methods).

For every experiment, ACA yielded two anti-correlated networks at the slowest time scale (Net. 1 and Net. 2) that coincided with those two networks apparent in Fig. 3a (right), validating the performance of the algorithm. ACA was applied at each timescale to obtain two anti-correlated populations (Pop. A and Pop. B) which were then compared with Net. 1 and Net. 2. At progressively faster timescales (right to left in Fig. 3f), Net. 1 and Net. 2 blended together until they were indistinguishable. This process was quantified through the entropy of the contingency table displayed in Fig. 3f (top left) that ascribes neurons’ belonging to one network and one population. The entropy monotonically increased from the slowest to fastest timescale (Fig. 3g). Altogether, these results demonstrate that the emergence of well-defined anti-correlated networks occurs progressively with slower timescales of neural dynamics. Figure S3 shows how the anti-correlations arise from the slow modulation of firing patterns across neurons.

### Anti-correlated networks are heterogeneous and spatially distinct

**Figure 4:**
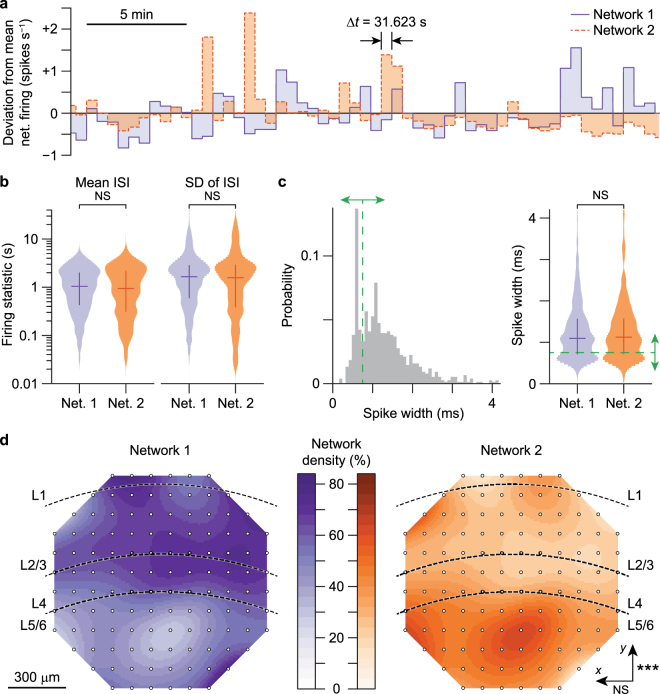
Anti-correlated cortical networks have preferential localization and are heterogeneously composed. (**a**) Fluctuations in mean neuronal activity of Networks 1 and 2 (*n* = 149 neurons from a single mouse) are anti-correlated over time. (**b**–**c**) Neurons (*n* = 764 neurons from six mice) in each anti-correlated network are equally heterogeneous in terms of (**b**) firing statistics and (**c**) spike widths. Bimodal spike width distributions identify neurons with narrow or broad spikes (green arrows), which putatively correspond to inhibitory and excitatory neurons, respectively. Crosses in violins in **b**–**c** show medians (horizontal lines) and first/third quartiles (vertical lines) of distributions. NS, not significant (Wilcoxon rank-sum test for equal medians, and Kolmogorov-Smirnov (KS) tests for equal distributions). (**d**) Neuronal density maps (*n* = 764 neurons from six mice) of the anti-correlated networks demonstrate differential bias to superficial and deep cortical layers (*P* = 1 × 10^−10^, KS test for equal distributions in neuron counts across *y*-axis), but not to the orthogonal dimension (*P* = 0.22, KS test for equal distributions in neuron counts across *x*-axis). NS, not significant; ***P < 0.001 (KS tests for equal distributions in neuron counts over the array electrodes).

The time series of the two competing networks at the slowest timescale were anti-correlated over time, as shown in Fig. 4a, and in the corresponding raster plot (Fig. S3a, middle) and binned spike trains (Fig. S3a, bottom). In Fig. 4a the peaks of activity in Network 2 correspond to transient increases in firing rate and are apparent as bright red spots in the binned spike trains in Fig. S3. Moreover, the slow shifts in network dominance which are apparent in Fig. S3 are also seen in Fig. 4a as changes in the sign of deviations from the mean firing rate. Although there were time intervals where both networks deviated in the same direction, there were other intervals where they deviated with greater magnitude in opposing directions, leading to the predominance of anti-correlated network activity. Furthermore, this effect was present at multiple bin widths, highly consistent across animals in independent slice preparations (Figs S1–S2), and disappeared when the binned spike counts were randomly shuffled (Fig. S1, bottom row). This suggests that the predominance of the anti-correlated activity cannot be attributed to a specific recording period, but rather that it is an intrinsic mode of operation of local cortical circuits.

However, there were no significant differences in the networks’ firing statistics: the means (*P* = 0.49, Wilcoxon rank-sum test; *P* = 0.11, Kolmogorov-Smirnov (KS) test for equal distributions) and standard deviations (*P* = 0.39, Wilcoxon rank-sum test; *P* = 0.15, KS test for equal distributions) of the ISI were similarly distributed between networks (Fig. 4b). These neuronal firing statistics and other spike features, such as spike width, have been used in establishing neuronal identities^24^. Narrower spikes putatively correspond to fast spiking, inhibitory interneurons while broader spikes correspond to slower spiking, excitatory projection neurons. Consistent with these characteristics, the distribution of widths was bimodal. However, the distribution of putatively excitatory and inhibitory neurons across experiments was not significantly different between networks (*P* = 0.81, Wilcoxon rank-sum test; *P* = 0.99, KS test for equal distributions), suggesting that each network was composed of both excitatory and inhibitory cells in similar proportions (Fig. 4c). Remarkably, however, these heterogeneous networks localized anatomically (Figs 4d and S4): one network was significantly biased to superficial cortical layers whereas the other network was biased to lower layers.

## Discussion

We have shown that spontaneous neuronal activity in isolated, neocortical circuits is highly diverse and organized into two heterogeneous networks with anti-correlated firing fluctuations, which are spatially distinct and most apparent at timescales on the order of several seconds. Local cortical circuits, such as those investigated here, are believed to be the building blocks for cognitive functions such as sensory processing and the generation of motor commands. In this framework, the anti-correlated networks observed in local circuits may indicate intrinsic modes of operation in the brain to encode or coordinate antagonistic behaviors.

The localization of slow transitions of neuronal firing to cortical anatomy is not surprising since multi-temporal neural phenomena^25^, as evidenced by Fig. 2, are often inextricably tied to multiple spatial scales of anatomical interaction^26^. Of note, while weak, sparse, and positive correlations existed at fast timescales/smaller bin widths (down to ∆*t* = 1 ms), correlations characteristic of two competing networks emerged at slow timescales/larger bin widths (∆*t* = 100 ms and longer). Cortical neurons are known to operate at the short timescales of monosynaptic inhibitory lags and synaptic decay kinetics, which may be optimal for information transmission and spiking coherence/reliability^27,28^. In other spontaneously active neural systems, neuronal networks or populations are often reported to exhibit interactions at these timescales but also show emergent correlation structure at slower timescales^29,30^. These slow interactions are to be expected when viewed in the functional context of behavior: the primary somatosensory cortex of the mouse is tied to whisker vibrations or multi-temporal vibrissa movements during various exploratory behavioral states^16,31–34^.

### Competing layers at behaviorally relevant timescales

In isolated local circuits, the spontaneously anti-correlated networks reported here correspond to predominantly superficial and deeper layers. The anatomical and operational distinction between superficial and deep layers is well established across cortical areas: through development^35^, in response to stimulation or the absence of stimulation^36–38^, and in different cortical states^39–42^. Furthermore, local field potential (LFP) studies have shown that gamma-band neuronal activity in superficial layers alternates with slower rhythms—alpha rhythms in the visual cortex of behaving monkeys^43^ and beta rhythms in hippocampal slices^44^—in deeper layers.

The anti-correlated networks reported here may be extended to a behavioral context when considering the distinct exploratory modes of the rodent, which shift between feedforward sensation and top-down control configurations on a timescale of seconds. These configurations correspond with active sensing and anticipatory behavioral states^15,16^ and are layer-specific^45^: early activation of deep layers correlates with anticipatory behavior, whereas early activation of superficial layers correlates with active sensing^17^. The rodent’s ability to switch between these behavioral modes is critical since each mode involves the mutually exclusive use of subcortical brain structures and peripheral resources.

### Spontaneous activity in cortical circuits

The temporal range over which the anti-correlated networks were observed (Fig. 3a and f) is particularly notable. The networks were readily detectable on the order of ∆*t* = 100 ms (corresponding to 10 Hz neuronal bursting/firing) but became increasingly distinct for larger ∆*t*. Furthermore, the number of connections or *edges* as determined by the adjacency matrices (Figs 3b and S2) reaches a maximal value on the order of ∆*t* = 1 s. This is quantified by the degree of the networks across timescales (Fig. 3d).

This span of timescales in cortical circuits encompasses two well-documented phenomena: LFP oscillations and Up/Down states. Synchronization of neighboring neurons is a longstanding prerequisite for the observation of LFP oscillations^46^; thus, in asynchronous populations like the ones reported here, it is possible that LFPs do not constructively summate. Consistent with this, we do not observe LFP oscillations in our raw recordings. Nevertheless, individual neurons may still burst or fire in the same frequency bands, a phenomenon we observe and report here.

On timescales of the order of 1 s, cortical networks are known to shift their constituent neurons between high and low states of excitability, commonly referred to as Up and Down states^47–49^. They are often associated with, but are not necessarily limited to, slow wave sleep *in vivo*^50,51^, and have been known to propagate across and localize in different cortical layers^52,53^. Previous *in vivo* and *in vitro* studies have demonstrated independent laminar operation—states present in lower layers but not upper layers—and interlaminar propagation during spontaneous cortical activity and network-wide Up states, confirmed by whole-cell recordings, in the case of feedforward sensory input^3,11,39^.

The detection of Up/Down states is ambiguous in extracellular multiunit recordings, since Up/Down states are defined by a neuron’s intracellular membrane potential. Although it is possible that synchronous Up/Down states mediate *intra*-network interactions, the asynchronous *inter*-network interaction we observe—that of two anti-correlated laminar networks—points to a larger spatiotemporal coordination of asynchronous cortico-cortical interaction.

### Mechanisms for slow network dynamics

Neuromodulation is a candidate mechanism that may account for slow fluctuations in firing. It is well known that the somatosensory cortex is densely innervated by subcortical neuromodulatory inputs^54^. However, slow firing fluctuations have been previously observed in cultures of dissociated cortical networks, in which neuromodulatory afferents are absent^55^, suggesting that neuromodulation is not necessary for the emergence of slow firing fluctuations. Instead, in dissociated cortical networks, intracellular calcium transients alone have been shown to trigger coordinated bursts on the timescale of several seconds^56^. Altogether, this suggests that neuronal circuitry alone is sufficient for the slow dynamics observed in acute brain slices, giving rise to anti-correlated networks through the recurrent connections between superficial and deep layers.

A general, perhaps more parsimonious explanation of the findings reported here is provided by dynamical systems theory, as presented by Haken in the celebrated context of synergetics^57^. The theory explains that any complex system of interacting elements operating at criticality will be dominated by collective modes with little attenuation, that is, with long time constants. In the presence of noise, which is ubiquitous in neural circuits^27,58,59^, collective modes fluctuate on the timescale given by their time constant. Moreover, the variance of their fluctuations is proportional to their time constant—a property of so-called Ornstein-Uhlenbeck processes^60^. In light of this theoretical framework, we note that the fluctuation scaling of neuronal firing shown in Fig. 2d (Taylor’s power law) is a clear signature of critical behavior, as it indicates that larger fluctuations correlate with slower firing rates. Finally, we note that the dominant spatiotemporal modes predicted by the theoretical framework of synergetics would correspond to the ongoing competition between superficial and deeper layers reported here.

## Methods

### Slice preparation

All experimental protocols were approved by the Institutional Animal Care and Use Committee of Case Western Reserve University and all experiments were performed in accordance with their guidelines. Spontaneous activity in acute cortical slices has been previously shown to be dependent on both the ionic composition and oxygenation of perfusion solutions^9^. We used the buffer solutions reported by the MacLean lab^10^ which allowed us to measure sustained, non-epileptogenic activity. A series of three artificial cerebrospinal fluid (aCSF) solutions were prepared for (1) brain extraction and cutting (4 °C); (2) incubation (30 °C); and (3) perfusion (31 °C). All solutions were cooled or warmed to their designated temperature before they were saturated with 95% O_2_–5% CO_2_. Temperature and oxygen measurements were made with a World Precision Instruments OxyMicro system with a MicroTip sensor (50 μm). Coronal slices (350 μm thickness) of primary somatosensory cortex (S1) were collected from juvenile (P13–P17) C57BL/6 mice. Animals were anesthetized with vapor isoflurane and decapitated with a guillotine. Brains were then submerged in ice-cold aCSF containing (in mM): 3 KCl, 26 NaHCO_3_, 1 NaH_2_PO_4_, 0.5 CaCl_2_, 2.5 MgSO_4_, 25 glucose, 123 sucrose. Brain slices were cut on a vibratome (Leica VT1200). All chemical salts and reagents were purchased from Fisher Scientific (Pittsburgh, PA) and Sigma-Aldrich (St. Louis, MO).

Slices were directly collected and transferred to the oxygenated incubation solution (56.0 kPa O_2_), containing (in mM) 123 NaCl, 3 KCl, 26 NaHCO_3_, 1 NaH_2_PO_4_, 2 CaCl_2_, 6 MgSO_4_, 25 glucose, to equilibrate for 30 minutes. Finally, a single slice was selected and placed into the recording chamber containing oxygenated aCSF (53.3 kPa O_2_), with (in mM) 123 NaCl, 3.5 KCl, 26 NaHCO_3_, 1 NaH_2_PO_4_, 1.2 CaCl_2_, 1 MgSO_4_, and 25 glucose. The entire thickness of the somatosensory cortex (~1 mm) was fixed to the 1.2 × 1.2-mm MEA perforated recording field with 15-mbar suction and the slice was continuously perfused with aCSF at 6.5 ml/min and 31 °C for 30 min before the recording was started.

### Microelectrode array recordings

High-density microelectrode arrays (MEAs) with 120 electrodes (100-μm pitch) and a MEA-2100 System (amplifier and digitizer) from Multichannel Systems (MCS, Reutlingen, Germany) were used to record from S1. The activity from the acute brain slice was recorded at a sampling frequency of 50 kHz with a resolution of 16 bits in the range of −2 mV to 2 mV, and two sequential hardware filters (2nd order 0.5-Hz high-pass filter; 1st order 10-kHz low-pass filter) were used to eliminate voltage offsets and drifts.

### Spike sorting

All processing and other data analyses were carried out in MATLAB (version 2016b; Mathworks, Natick, MA). The recordings from all channels were digitally band-pass filtered from 80−2,000 Hz with a 3rd order Butterworth filter. Spikes were detected as events falling outside of positive and negative potential thresholds defined during 1-s windows as ± 4 × standard deviation. Spike sorting was conducted with Wave_clus, a freely available wavelet-based algorithm^18^. Up to 6 neurons could be detected in a single channel. Spike trains that appeared in more than one electrode with < 1 ms delays were considered to come from the same neuron; the spike train with the greatest spike amplitude was kept and the rest were eliminated. From each spike sorted neuron, we computed the mean waveform and determined its amplitude and width at the baseline potential.

### Firing statistics

Interspike interval (ISI) series were computed as the temporal differences between consecutive spikes from a single given neuron. Neurons firing at an average rate of less than 10 spikes/min throughout the experiment (37 ± 5 neurons, 19% ± 2%; mean ± s.e.m. across seven experiments) were eliminated from all further analyses.

### Spike trains as count processes—binning

For the remaining statistical analyses, we treated the spike trains as count processes. We started with the data as binary spike trains at the original sampling frequency of 50 kHz, whereby every detected spike was considered a single event marked as a 1 and the absence of a spike as a 0. We then binned these binary spike trains by dividing the time axis into consecutive bins of width ∆*t*, and counting the spikes in each bin. Thus, for each neuron at a given ∆*t*, we obtained a time series of the spike count. Then, by varying ∆*t* over 20 evenly spaced steps (between 10^−3^ s and 10^1.5^ s) on a logarithmic scale, we were able to perform our analyses over multiple time scales.

### Spike train spectra and auto-correlograms

Both the spectrum and auto-correlogram of a spike train characterize its temporal structure. We binned the spike trains at ∆*t* = 1 ms and computed their spectra with the multitaper method as described previously^61^, with bandwidth parameter *W* = 1 mHz, signal duration *T* = 600 s, and 300 Slepian functions. We also computed and plotted the normalized auto-correlogram of the binned spike trains for multiple selected timescales ∆*t*. For further processing, each binned signal was mean-subtracted and divided by the standard deviation of the resulting signal.

### Connectivity and graph analysis

For a given experiment, at every timescale ∆*t*, pairwise Pearson correlations between the binned spike counts of neurons were used to determine the correlation matrix ***C*** across neurons. Statistical significance of pairwise correlations was determined by means of random permutations conducted as follows. The time series of the binned, spike counts of each neuron in a pair were randomly permuted and correlated with each other. This process was conducted 3,000 times to generate a distribution of correlations for that pair. For each pair, *P* values (left-tailed probabilities) of their empirical correlation were then computed relative to their respective distribution. Correlations with (i) *P* value ≤ 0.005 or (ii) *P* value ≥ 0.995 were considered highly significant; criterion (i) identifies significant *negative* correlations, and criterion (ii) significant *positive* correlations. Binary adjacency matrices ***A*** were then constructed from ***C*** at each timescale, with an entry ***A***_*ij*_ equal to 1 if ***C***_*ij*_ met either criterion (i–ii), and ***A***_*ij*_ equal to 0 otherwise. Each matrix ***A*** defines an undirected, unweighted graph of functional neuronal connectivity: an element ***A***_*ij*_ equal to 1 indicates a functional connection between neurons *i* and *j*, which represents an edge between nodes *i* and *j* in the graph. Diagonal elements ***A***_*ii*_ were set to 0, thereby disallowing self-connectivity (i.e. edges between a node *i* and itself). Neurons that had no functional connections to any other neuron across all timescales (28 ± 8 neurons, 19% ± 6%; mean ± s.e.m. across six experiments) were eliminated for further analyses.

Graph theoretic measures (as displayed in Fig. 3c–e) were computed using standard methodology. The largest connected partition at a given timescale was determined by summing the rows of ***A*** and eliminating every neuron whose corresponding sum was equal to 0; the remaining neurons thus formed a fully connected graph. The largest partition size (Fig. 3c) was then computed as the number of neurons in this partition divided by the total number of neurons in the original adjacency matrix ***A***. The degree *D*_*i*_ of a given neuron *i* in a graph comprising *n* neurons was computed as the number of significant connections to other neurons. The maximum degree normalized to network size (Fig. 3d) was determined as max(*D*)/*n*. The assortativity coefficient (Fig. 3e) was calculated as the Pearson correlation coefficient of degree between every pair of connected neurons.

### Anti-correlated component analysis

Because two anti-correlated networks are apparent across multiple timescales, we sought to rigorously identify a pair of anti-correlated networks at each timescale and determine their consistency. To this end, we developed an algorithm, which we call anti-correlated component analysis (ACA). Let ***z***(*t*) be the column vector where the *i*-th component is the *z*-score of the spike count between time *t* and *t* + ∆*t*. ***C***, the correlation matrix as defined above, is ${\boldsymbol{C}}=\langle {\boldsymbol{z}}(t){{\boldsymbol{z}}}^{T}(t)\rangle$, where $\langle \mathrm{...}\rangle$ represents averaging in time. The anti-correlated components ***u*** and ***v*** are the two directions in the *n*-dimensional space (*n* is the number of neurons) which minimize the product of the projected *z*-scores, that is, the cost function:

1 $$L=\langle {{\boldsymbol{u}}}^{T}{\boldsymbol{z}}(t){{\boldsymbol{z}}}^{T}(t){\boldsymbol{v}}\rangle ={{\boldsymbol{u}}}^{T}\langle {\boldsymbol{z}}(t){{\boldsymbol{z}}}^{T}(t)\rangle {\boldsymbol{v}}={{\boldsymbol{u}}}^{T}{\boldsymbol{C}}{\boldsymbol{v}}$$

In addition, the minimization is subject to the following constraints: ${\Vert {\boldsymbol{u}}\Vert }^{2}+{\Vert {\boldsymbol{v}}\Vert }^{2}=1$ and ***u***, ***v*** ≥ 0. Together, these conditions define a constrained saddle-point minimization problem in a 2*n*-dimensional space. To see this, consider the expanded matrix $$\tilde{{\boldsymbol{C}}}=[\begin{array}{cc}{\bf{0}} & {\boldsymbol{C}}\\ {\boldsymbol{C}} & {\bf{0}}\end{array}]$$ and vector $${{\boldsymbol{x}}}^{T}=[\begin{array}{cc}{\boldsymbol{u}} & {\boldsymbol{v}}\end{array}]$$; then, the cost function $L$ in (1) can be re-written as:

2 $$L=\frac{1}{2}{{\boldsymbol{x}}}^{T}\tilde{{\boldsymbol{C}}}{\boldsymbol{x}},$$

and the constraints as ${\Vert {\boldsymbol{x}}\Vert }^{2}=1$ and ${\boldsymbol{x}}\ge 0$. Equation (2) represents a quadratic form in a 2*n*-dimensional space; in particular, it is a hyperbolic saddle centered at the origin, and the first constraint is a hypersphere delimiting that saddle. The intersection of the saddle and the sphere is not necessarily a convex manifold. However, the additional constraint to the 2*n*-dimensional hyperoctant (${\boldsymbol{x}}\ge 0$), which removes the ambiguity of the cost function’s sign, leads to a unique solution if the saddle is not degenerate (no repeated eigenvalues of $\tilde{{\boldsymbol{C}}}$). Because of the presence of noise, the covariance matrices from our dataset are not degenerate and therefore, neither is $\tilde{{\boldsymbol{C}}}$, and the solutions are indeed unique within the numerical tolerance of the optimization. The minimization algorithm was implemented in MATLAB using the function fmincon from the optimization toolbox. The code is available in the repository of Mathworks® File Exchange^23^.

Once the solution is obtained, if the *i*-th element (neuron) of ***u*** is negligible (numerically zero), then the *i*-th element of ***v*** will be significantly larger than zero and vice versa. Thus, the non-zero elements in each network are non-overlapping, and therefore represent mutually exclusive networks. Note that this minimization procedure will generally lead to projected *z*-scores ***u***^*T*^***z***(*t*) and ***z***^*T*^(*t*)***v*** that are, over time, maximally anti-correlated, representing the maximally anti-correlated activity between the two networks identified by ***u*** and ***v***.

Note that since ACA is informed by the correlation matrix, it is sensitive to both positive and negative correlations in the dataset, and so are the two anti-correlated networks: all neurons in the same network are positively correlated with each other and are negatively correlated with the neurons in the other network. In its current form, ACA is limited to finding the two most anti-correlated components in a dataset. Future work will investigate non-trivial generalizations to obtain higher-order components.

### Entropy and kappa measures

Observations of correlation matrices ***C*** across timescales (as in Fig. 3a and Movie S2) motivated an investigation of the blending of the two networks at faster timescales. After performing ACA on ***C*** at every ∆*t*, the separation between anti-correlated networks was most apparent at the slowest timescale, ∆*t* = 31.623 s, as seen in Fig. 3a (right); the two anti-correlated networks at this timescale thus defined Network 1 and Network 2. Because this structure blurs at progressively faster timescales, we needed to compare the anti-correlated networks returned by ACA at every timescale (termed Population A and Population B) with Network 1 and Network 2. This way, at each timescale, we obtained a 2 × 2 contingency table by counting the number of neurons with each pairing of network label (1 or 2) and population label (A or B) (Fig. 3f, top left).

Consequently, the entries in this contingency table encode the answers to two yes/no questions: (i) Does a given neuron belong to Net. 1 or Net. 2? and (ii) Does a given neuron belong to Pop. A or Pop. B? The strength of the association between the answers to these two questions across all *n* neurons is elegantly captured by the Shannon entropy *H*, which is computed as $H=-\sum _{k}{p}_{k}{\mathrm{log}}_{2}{p}_{k}$ (and is measured in bits), where *p*_*k*_ = *n*_*k*_/*n* is the proportion of neurons with a given pairing *k* of network label and population label.^62^

Additionally, we quantified the significance of the agreement between the network and population labels at each timescale using Cohen’s *κ*, a measure of inter-rater agreement between two binary classification schemes^63^. Per timescale, the unweighted *κ* statistic and its *P* value were computed for each experiment separately, and in Fig. 3f the averages of these *κ* and *P* values are reported.

## Electronic supplementary material

Supplementary Figures

Supplementary Video 1

Supplementary Video 2
