# Supplementary material for: Anti-correlated cortical networks arise from spontaneous neuronal dynamics at slow timescales
Source: Sci Rep. 2018 Jan 12;8:666. doi: 10.1038/s41598-017-18097-0 (PMC5766587; doi:10.1038/s41598-017-18097-0)
Supplement: Supplementary file 1 — Supplementary Figures [file 41598_2017_18097_MOESM1_ESM.pdf]

SUPPLEMENTARY INFORMATION

Anti-correlated cortical networks arise from spontaneous neuronal dynamics at slow timescales

Nathan X. Kodama[1], Tianyi Feng[1], James J. Ullett[1], Hillel J. Chiel[2], Siddharth S. Sivakumar[1] & Roberto F. Galán[1,*]

[1]Department of Electrical Engineering and Computer Science, Case Western Reserve University, Cleveland, Ohio 44106, USA.
[2]Department of Biology, Case Western Reserve University, Cleveland, Ohio 44106, USA.

***Corresponding Author**
Roberto Fernández Galán, PhD (rfgalan@case.edu).

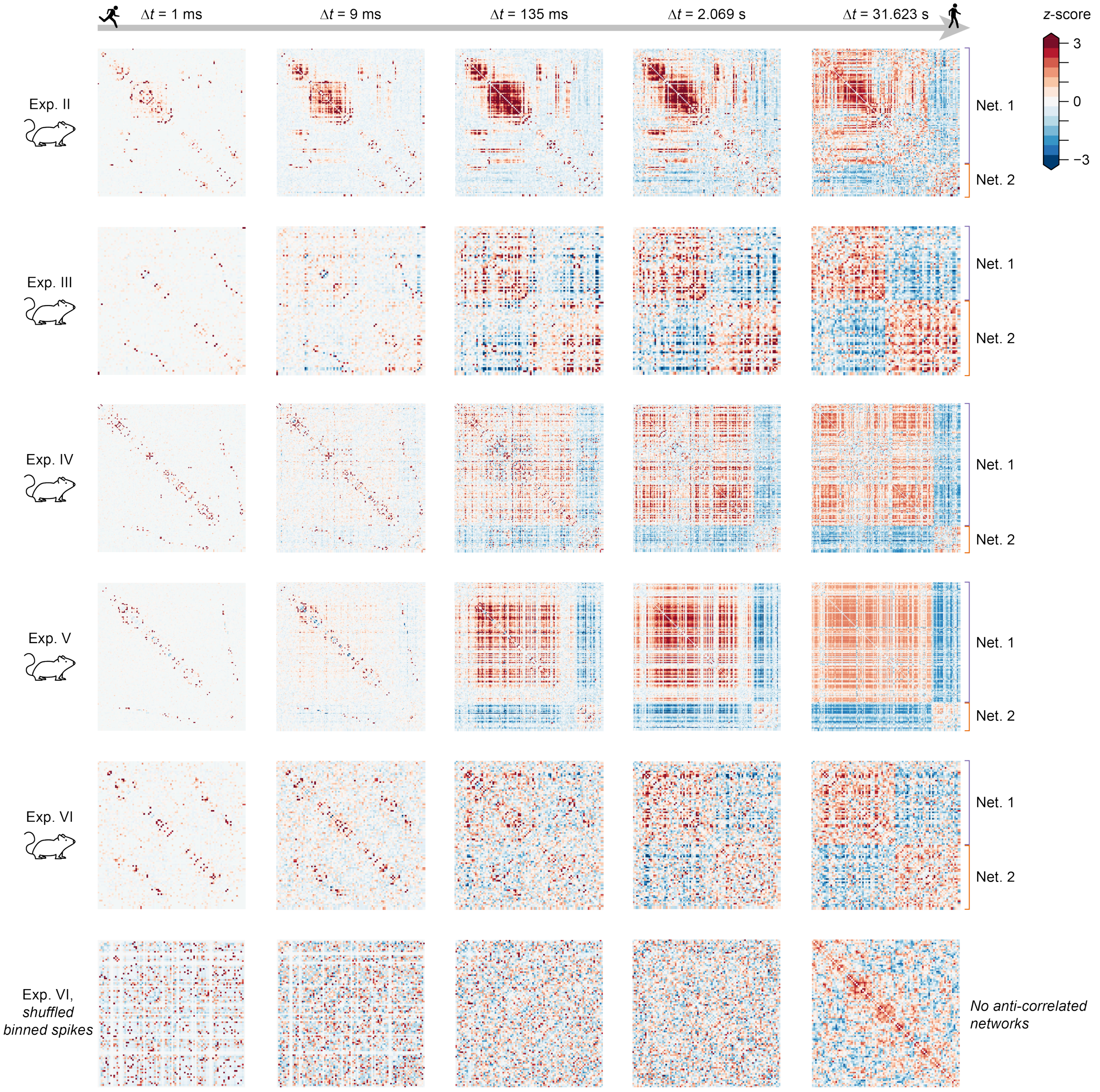

Figure S1. Correlation matrices of binned spike counts across experiments. Correlation matrices of binned spike counts from five additional experiments (Exp. II–VI), at five selected timescales (Δt). Correlation values were normalized by their z-score at each timescale. At the slowest timescale (Δt = 31.623 s), correlation matrices clearly show two mutually exclusive, anti-correlated networks (Net. 1, Net. 2) in each experiment. Bottom row, correlation matrices of binned spike counts shuffled in time for each neuron in Exp. VI; no anti-correlated networks are apparent from these correlation matrices.

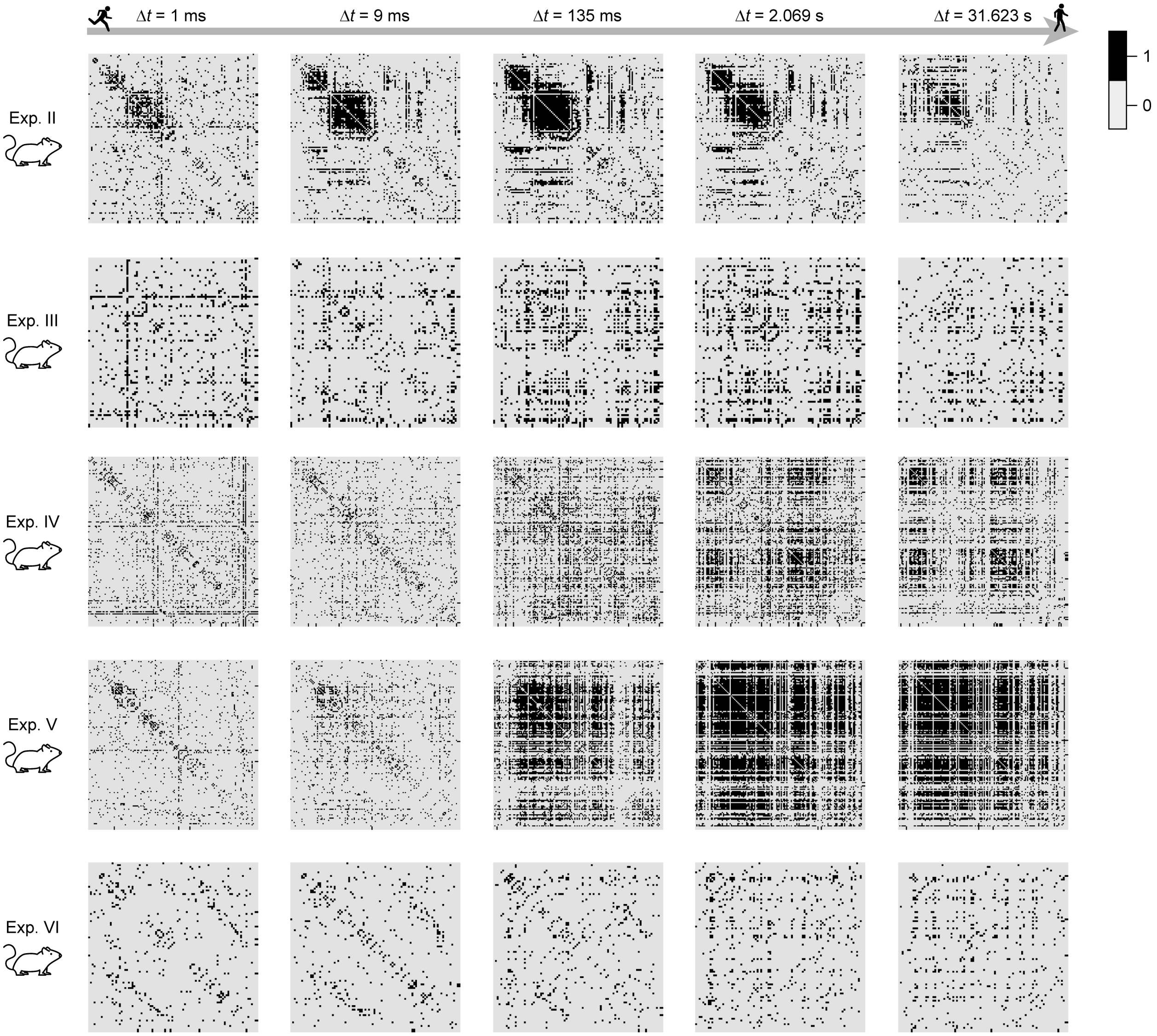

Figure S2. Binary adjacency matrices across experiments. Adjacency matrices were computed from correlation matrices (shown in Fig. S1) as highly significant (1) or non-significant (0) pairwise correlations at each timescale Δt, for five additional experiments (Exp. II–VI). Functional connections are stronger and denser at slower timescales.

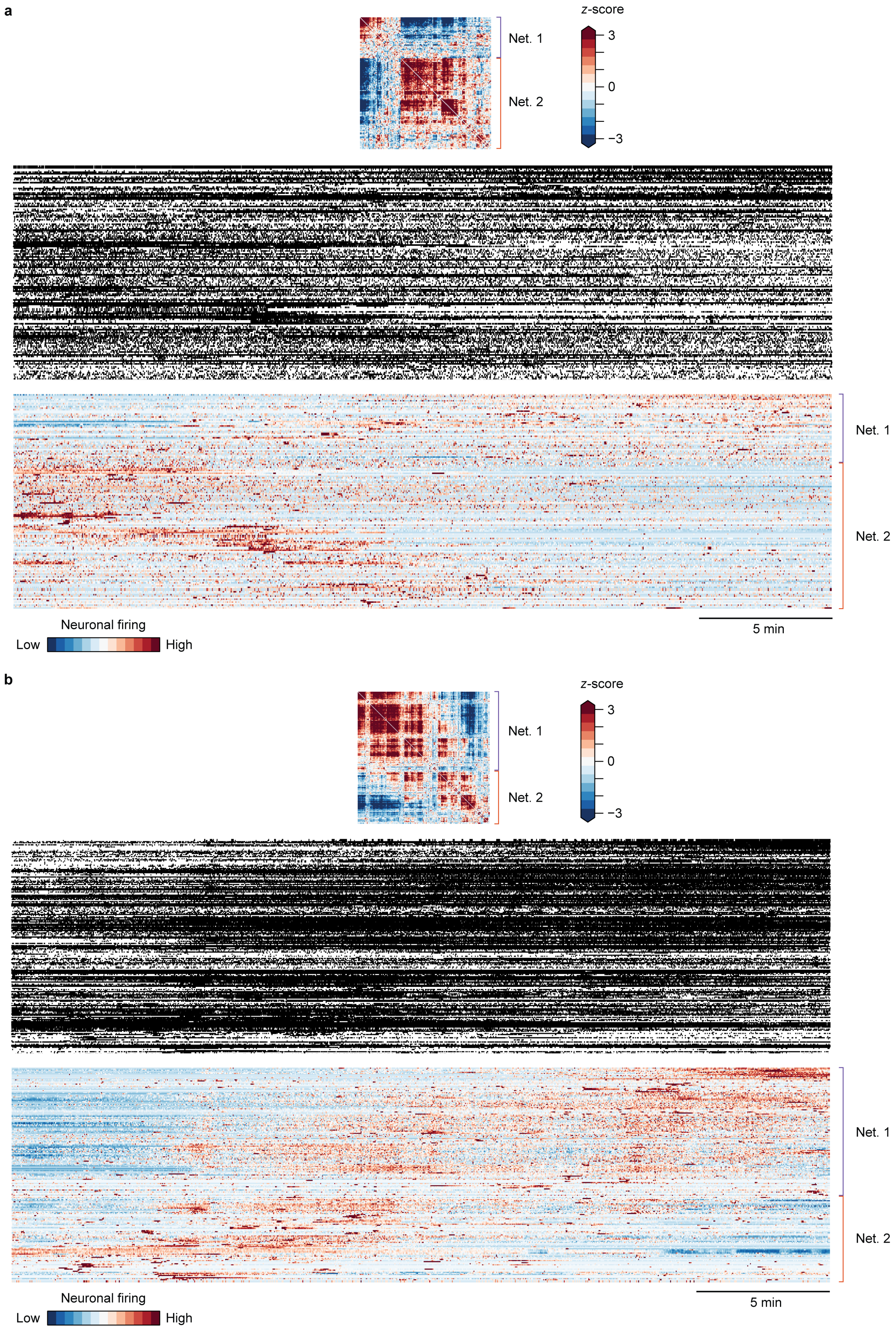
a
Net. 1
Net. 2
z-score
3
0
−3
Net. 1
Net. 2
Neuronal firing
Low
High
5 min
b
Net. 1
Net. 2
z-score
3
0
−3
Net. 1
Net. 2
Neuronal firing
Low
High
5 min

Figure S3. Anti-correlated networks are visible in population spiking patterns. (**a**) Top, correlation matrix (normalized by its *z*-score) of spike counts binned at $\Delta t = 31.623$ s; middle, raster plot of neurons; and bottom, binned time series of neuronal firing (normalized by each neuron's *z*-score), of two anti-correlated networks (Net. 1, Net. 2; $n = 145$ neurons from a single mouse) observed over 30 min. (**b**) Similar plots for another mouse ($n = 201$ neurons) over 30 min.

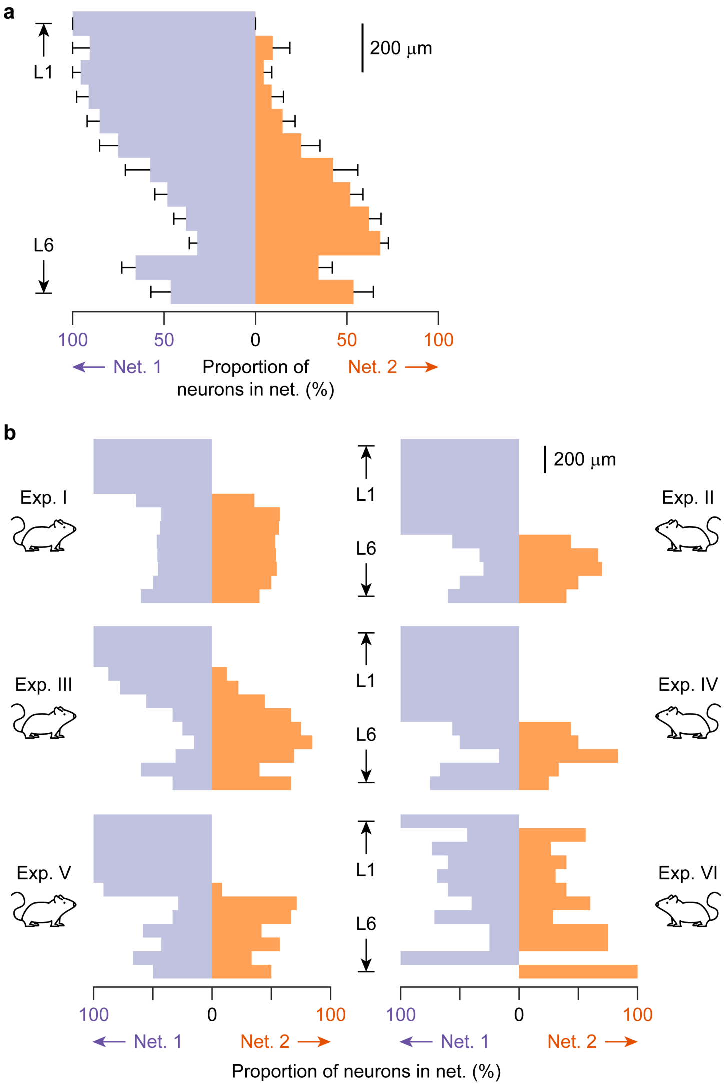

Figure S4. Preferential localization of anti-correlated networks across experiments. (**a**) Proportions of neurons coming from each anti-correlated network (Net. 1, Net. 2) at different depths from pia, averaged across experiments ($n = 764$ neurons from six mice). Error bars show s.e.m. across six experiments. L1, cortical layer 1; L6, cortical layer 6. (**b**) Per experiment (Exp. I–VI), network proportions at different depths demonstrate preferential localization of Net. 1 to superficial layers and of Net. 2 to deeper layers.

SUPPLEMENTARY MOVIE CAPTIONS

Movie S1. Auto-correlograms of spike trains across multiple timescales Δt. Oscillations of different amplitudes and frequencies appear at different timescales across neurons. Each trace corresponds to one of the three representative neurons displayed in Figs. 1d, 2a, and 2c. Throughout the animation, Δt progressively decreases from 31.623 s to 1 ms. The *x*- and *y*-axes simultaneously zoom in to emphasize the oscillatory nature of the auto-correlograms across timescales.

Movie S2. Correlation matrices of the binned spike counts across timescales. Throughout the animation, Δt progressively increases from 1 ms to 31.623 s in logarithmically spaced increments. The row- and column-ordering of the correlation matrix $\boldsymbol{C}$ (n = 149 neurons) across timescales is consistent. Correlation values were normalized by their *z*-score. From fast to slow timescales, the emergence and consolidation of two anti-correlated networks (Networks 1 and 2) is apparent.
